# Based on Network Pharmacology and Gut Microbiota to Explore the Underlying Mechanism of Huangqi Gegen Decoction for Treating Metabolic‐Associated Fatty Liver Disease

**DOI:** 10.1002/fsn3.71133

**Published:** 2025-11-09

**Authors:** Linzi Li, Yuxian Zhang, Cong Zhou, Chengxiong Yang, Chunkai Rao, Daoping Zhang, Xu Ai, Xianqiang Shao, Jing Zhou, Yan Yang, Shanshan Lei

**Affiliations:** ^1^ Jingmen Central Hospital Jingmen Hubei China; ^2^ Jingmen Central Hospital Affiliated to Jingchu University of Technology Jingmen Hubei China; ^3^ School of Medicine & Shaanxi University Engineering Research Center of Development and Utilization of Qinba Traditional Chinese Medicine Resources AnKang University AnKang Shaanxi Province China; ^4^ Jingchu University of Technology Jingmen Hubei China; ^5^ Hubei Gewa Food Co., Ltd Zhongxiang Hubei China; ^6^ Department of Medicine Zhejiang Academy of Traditional Chinese Medicine Hangzhou Zhejiang China

**Keywords:** gut microbiota, Huangqi Gegen Decoction, MAFLD, network pharmacology, pharmacological mechanism

## Abstract

Huangqi Gegen Decoction (HQGG) is composed of *Astragali Radix* and *Puerariae Lobatae Radix,* both of which are recognized as therapeutic agents and edible supplements, and has been reported as a potential treatment for metabolic‐associated fatty liver disease (MAFLD). This study employed a comprehensive approach integrating network pharmacology, molecular docking, in vivo experimentation, and gut microbiota analysis to assess the efficacy of HQGG in treating MAFLD and to explore its molecular mechanisms. The results indicated that HQGG has significantly ameliorated hepatocellular injury, decreased liver inflammation, and oxidative stress in the HFD‐induced MAFLD model rats. The network pharmacology identified 21 bioactive compounds, predicted 238 potential targets, and uncovered that the main active components of HQGG may regulate PTGS2, AKT1, MAPK1, JUN, and PPARG to confer their alleviating effects against MAFLD. HQGG also acted on various signaling pathways to treat MAFLD, such as the AGE‐RAGE signaling pathway in diabetic complications, lipid and atherosclerosis, non‐alcoholic fatty liver disease, and the IL‐17 signaling pathway. The in vivo experiments revealed that HQGG may achieve the effect of anti‐MAFLD by regulating the PPAR‐γ/NF‐κB signaling pathway. The results of gut microbiota analysis showed that HQGG could modulate the species structure and abundance, regulating gut microbiota imbalance of MAFLD rats. Overall, the results disclosed that HQGG can affect bacterial diversity and community structures in the gut and the PPAR‐γ/NF‐κB signaling pathway to treat MAFLD. This study systematically elucidated the potential mechanism of HQGG in treating MAFLD, providing a theoretical basis for the development and application of HQGG as a functional food for preventing MAFLD.

## Graphical abstract

This study employed a comprehensive approach integrating network pharmacology, molecular docking, in vivo experimentation, and gut microbiota analysis to evaluate the efficacy of HQGG in the treatment of MAFLD and to explore its molecular mechanisms. Our findings suggest that HQGG can affect bacterial diversity and community structures in the gut and the PPAR‐γ/NF‐κB signaling pathway to treat MAFLD.

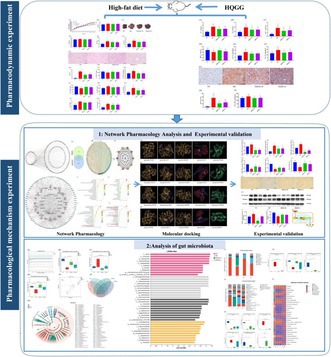

**Abbreviations**
BP: biological processes
CC: cellular components
C‐T: compound‐target
C‐T‐P: compound‐target‐pathway
DL: drug‐likeness
GO: gene ontology
H&E: hematoxylin–eosin
HFD: high‐sucrose‐fat diet
HQGG: Huangqi Gegen Decoction
KEGG: Kyoto Encyclopedia of Genes and Genomes
MAFLD: metabolic‐associated fatty liver disease
MF: molecular functions
OB: oral bioavailability
PPI: protein–protein interaction
TCM: Traditional Chinese Medicine
TCMSP: Traditional Chinese Medicine Systems Pharmacology

## 1 Introduction

MAFLD is the most prevalent chronic liver condition globally. Without timely intervention, MAFLD can not only lead to liver fibrosis, cirrhosis, and liver failure, but may also promote the occurrence of extra‐hepatic metabolic diseases (such as cardiovascular, cerebrovascular diseases, etc.) (Ng et al. 2022; Pipitone et al. 2023), rendering the condition a significant global public health problem. According to one epidemiological survey, the prevalence of MAFLD is currently 25% (Zhen et al. 2021; Hou et al. 2011), a value which is increasing year by year and trending toward lower ages (Zhu et al. 2015). At present, specific therapeutic drugs for MAFLD have become a hot topic; as an essential part of complementary and alternative therapy, Traditional Chinese medicine (TCM) has been reported as a potential treatment for MAFLD (Deng et al. 2022; Zhou, Zhang, You, et al. 2022).

The gut‐liver axis represents a bidirectional interaction between the gut microbiota and the liver, and has emerged as a crucial player in the pathogenesis of MAFLD (Albillos et al. 2020). The gut microbiota regulates and stabilizes through a complex system of interactions involving metabolic, immune, and neuroendocrine crosstalk between them. It plays a vital role in the gut and liver dialogue (Kho and Lal 2018). Studies have shown that TCM can not only directly act on disease‐related targets to exert its effects, as well as treat diseases by influencing gut microorganisms, regulating their metabolism, and transforming Chinese medicinal compounds (Bao et al. 2022; Yue et al. 2022). This provides a new scientific theoretical basis for the mechanism research of TCM.

The Huangqi Gegen decoction (HQGG) is derived from the book “Zhengzhi Huibu”, made up of two Chinese herbal medicines, namely *Astragali Radix* and *Puerariae Lobatae Radix* that can be used for both food and medicine. Modern pharmacological studies have demonstrated that HQGG has various effects, including reducing insulin resistance (Wei et al. 2022), regulating dyslipidemia (Zhou et al. 2021), and having an anti‐inflammatory effect (Cong et al. 2021). These suggest that HQGG may exert anti‐MAFLD effects through multiple mechanisms, but the mechanism by which HQGG treats MAFLD remains unclear.

**FIGURE 1:**
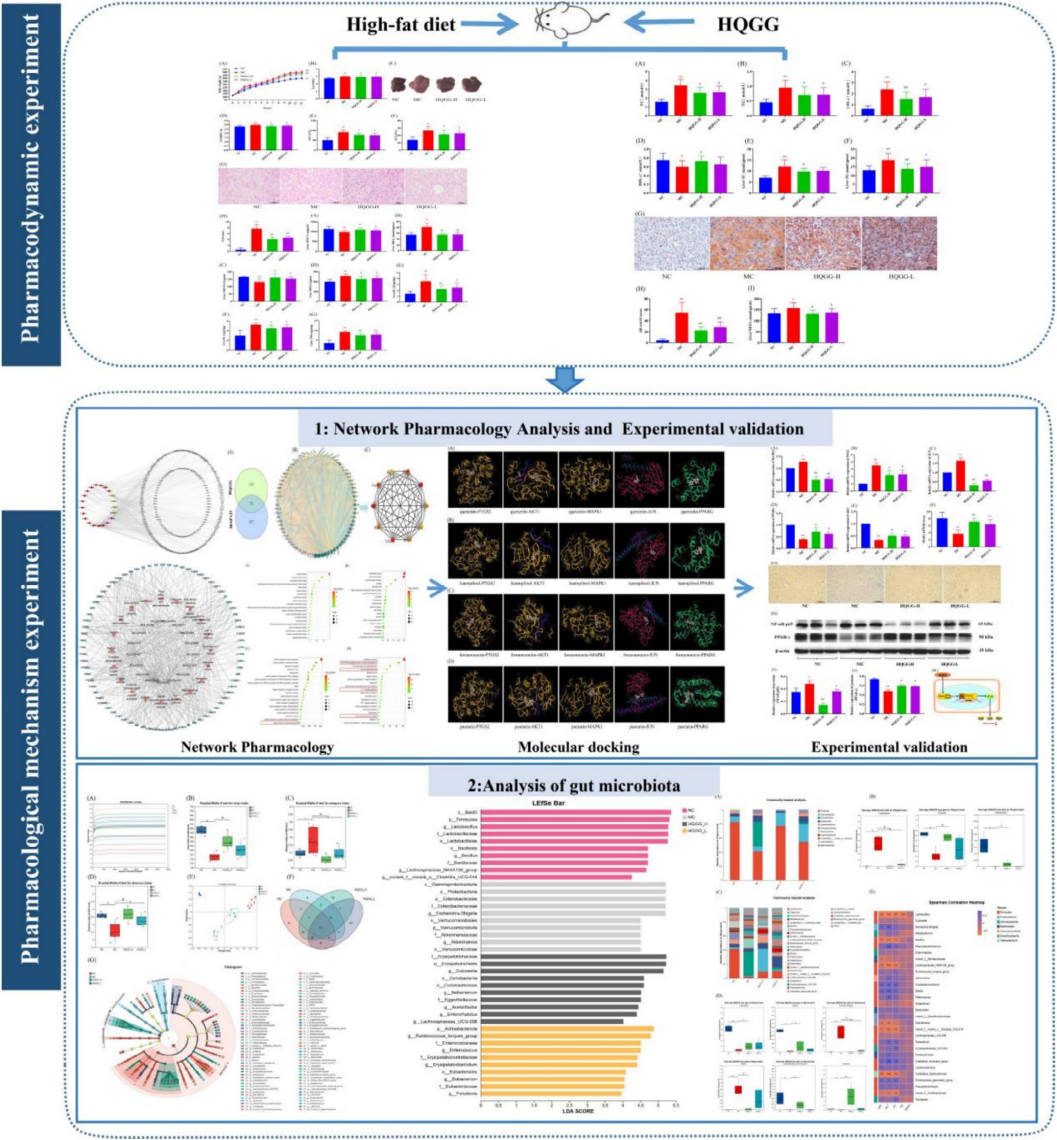
The graphical abstract of HQGG beneficial effect on MAFLD.

Network pharmacology is a multi‐disciplinary product of traditional pharmacology, biochemistry, and systems biology, which systematically analyzes the intricate relationship between drugs and diseases through a holistic perspective (Boezio et al. 2017). The network pharmacology research strategy aligns with the multi‐component, multi‐target, and multi‐pathway treatment characteristics of TCM, which provide references and ideas for further research on the pharmacological mechanism of TCM (Zhao et al. 2023). This study utilized network pharmacology and gut microbiota analysis to investigate the potential mechanism of HQGG in treating MAFLD, and providing a theoretical basis for the potential development and application of HQGG as functional food for the prevention of MAFLD (Figure 1).

## 2 Materials and Methods

### 2.1 Animal Experiments

#### 2.1.1 Preparation of HQGG

The HQGG herbs were obtained from Jingmen Central Hospital, which are in line with the requirements of the 2020 edition of the Chinese Pharmacopeia and strictly implemented following national execution standards. The dosage proportions of *Astragali Radix* (No. 230101, Gansu, China) and *Puerariae Lobatae Radix* (No. 230215, Hubei, China) are 2:1. We decocted all HQGG crude herbs with tenfold volume of distilled water for 2 times, the first time for 1.5 h, while the second time for 2 h, and then collected the decoctions after filtration to prepare the HQGG water extract. Finally, we used a rotary evaporator to concentrate so that the final concentration of the extract was 0.6 g/mL and stored at 4°C for use.

#### 2.1.2 Materials and Reagents

TC (230424202), TG (230328101), HDL‐c (230403101), ALT (231106101), and AST (231125201) in serum biochemical reagents kits were purchased from Ningbo Meikang Biotechnology Co. Ltd. (Zhejiang, China). MDA (20230120), SOD (20230125), GSH (20231217), and ROS (20231225) were purchased from JianCheng Bioengineering Institute, Nanjing, China. IL‐1β (1162065618), IL‐6 (1322057603), and TNF‐α (2312064617) ELISA kits were obtained from mlbio Biotechnology Co. (Shanghai, China). Hematoxylin–eosin (HE) dye solution (J23D9Y78310) was obtained from Shanghai Yuanye Bio‐technology Co. Ltd. (Shanghai, China). Primary antibodies against PPAR‐γ (AB_2793477, CPA4778), NF‐κB (AB_3071487, CPA7247), and β‐actin (AB_2750915, CPA9121) were purchased from Cohesion BIOSCIENCES (Suzhou, China).

#### 2.1.3 Animals and Experimental Design

SD rats (*n* = 32) provided by the Animal Supply Center of Zhejiang Academy of Medical Sciences (Hangzhou, China). Animal procedures were performed following the Regulations of Experimental Animal Administration and were approved by the Experimental Animal Welfare Ethics Committee of Zhejiang Academy of Traditional Chinese Medicine (Approval No.: Zhejiang Research Institute of Animal Ethics No. [2023]048). Feeding rats with high‐sucrose‐fat diets (HFD) to establish the MAFLD model is a mature MAFLD modeling method; the HFD consists of a normal diet of 78.5%, fructose 10%, edible lard 10%, cholesterol 1.25%, and bile salts 0.25%.

According to body weight, the SD rats were randomly assigned to 4 groups (*n* = 8): (1) normal control group (NC); (2) MAFLD model group (MC); (3) HQGG‐H treatment group (6 g/kg/d, P.O.); (4) HQGG‐L treatment group (3 g/kg/d, P.O.). Throughout the experiment, the NC rats were given a basic diet, whereas the other 24 rats were fed HFD. Both the NC and MC groups received corresponding distilled water via intragastric administration, while the HQGG treatment groups were given different doses of HQGG (3 g/kg/d and 6 g/kg/d, P.O.). After 12 weeks of treatment, the rats were fasted for 12 h before blood was collected through the orbital vein and were anesthetized by intraperitoneally injecting 2% pentobarbital sodium at an injection dose of 0.3 mL/100 g.

Following the experimental procedures, rat livers were weighed, with the largest hepatic lobes being preserved in 4% paraformaldehyde fixative solution, while the remaining liver tissues were cryopreserved at −80°C for subsequent analyses. We strictly followed humane endpoint criteria throughout the animal experiments. Rats were humanely euthanized by CO_2_ inhalation following isoflurane anesthesia. The death was validated by confirming cardiac and respiratory arrest. The mouse euthanasia was executed by Shanshan Lei.

#### 2.1.4 Detection of Biochemical Indicators

Following 12‐week treatment, whole blood samples were collected from the ophthalmic venous plexus, and serum fractions were extracted for subsequent biochemical analysis. The serum was then separated to measure the biochemical indicators of TC, TG, HDL‐c, ALT, and AST by automatic biochemical analyzer (Hitachi 7020, Japan); the serum LDL‐c was calculated using Friedewald's formula LDL‐c = TC ‐ (HDL‐c + TG/2.2). In addition, the levels of TC, TG, and NEFA in liver tissues were measured by commercial kits (JianCheng Bioengineering Institute, Nanjing, China).

The levels of oxidative stress indicators (MDA, SOD, GSH, and ROS) in liver tissues were also measured using commercial kits (JianCheng Bioengineering Institute, Nanjing, China). The concentration of IL‐1β, IL‐6, and TNF‐α in the liver was examined by ELISA (mlbio Biotechnology Co., Shanghai, China). All operations are carried out strictly following the manufacturer's operating procedures.

#### 2.1.5 Histological Analysis

Hepatic specimens underwent standardized histological processing involving immersion‐fixation in 4% paraformaldehyde, followed by paraffin embedding. Serial microtome sectioning at 3 μm thickness generated tissue slices for routine histological evaluation through hematoxylin and eosin (H&E) staining protocols. Cryosections of the liver were stained with 0.2% Oil Red O to visualize lipid deposition. The tissue sections were then examined under a biological microscope (Olympus BX43, Japan) and analyzed using Image‐Pro Plus software.

#### 2.1.6 Immunohistochemistry (IHC) Staining Observation

We used IHC to assess PPAR‐γ expression in the liver. Immunostaining protocols involved sequential incubations: primary antibody against PPAR‐γ, followed by secondary antibody HRP‐conjugated goat anti‐rabbit IgG. The tissues were observed under the biological microscope, and the Image‐Pro Plus analysis software was used to analyze the integrated optical density (IOD) of the positive area in the microgram semi‐quantitatively to evaluate the protein expression level.

#### 2.1.7 Real‐Time Polymerase Chain Reactions (RT–PCR)

Total RNA was extracted from liver tissue with TRI‐zol reagent and reversed transcribed. Real‐time polymerase chain reactions were performed according to the protocol provided by Sangon Biotech, detecting the mRNA levels of PTGS2, AKT1, MAPK1, JUN, and PPARG in liver tissue samples using β‐actin as the internal reference gene, and the fold change for each target gene was calculated using the 2 − ΔΔCt method, reduplicated 3 times.

Primer sequences were as follows: AKT1: forward 5′‐CACAGGTCGCTACTATGCCATGAAG‐3′ and reverse 5′‐GCAGGACACGGTTCTCAGTAAGC‐3′; PTGS2: forward 5′‐CACATTTGATTGACAGCCCACCAAC‐3′ and reverse 5′‐AGTCATCAGCCACAGGAGGAAGG‐3′; PPARG: forward 5′‐CCATCGAGGACATCCAAGACAACC‐3′ and reverse 5′‐GTGCTCTGTGACAATCTGCCTGAG‐3′; JUN: forward 5′‐GGAAACGACCTTCTACGACGATGC‐3′ and reverse 5′‐GGAGGTGCGGCTTCAGATTGC‐3′; and MAPK1: forward 5′‐TGAAGACACAGCACCTCAGCAATG‐3′ and reverse 5′‐GGTGTTCAGCAGGAGGTTGGAAG‐3′; β‐actin: forward 5′‐CGTAAAGACCTCTATGCCAACAC‐3′ and reverse 5′‐CGGACTCATCGTACTCCTGCT‐3′.

#### 2.1.8 Western Blot Analysis

The proteins from liver tissue were extracted by lysis buffer, and equal amounts of protein (20 μg) were separated by 12% sodium dodecyl sulfate‐polyacrylamide gel electrophoresis (SDS‐PAGE) and transferred onto the 0.45 μm PVDF membrane. The membranes were subjected to optimized immunoblotting procedures: initial blockade with 5% BSA for 60 min at 37°C, then incubated with 1:1000 diluted homologous primary antibodies (PPAR‐γ, NF‐κB, and β‐actin) in PBST with overnight incubation at 4°C. Post‐primary antibody incubation, three rigorous washing cycles were executed. The washed membrane was subsequently incubated with an HRP‐conjugated secondary antibody (diluted at a ratio of 1:5000) for 2 h at room temperature. The blotted protein bands were detected using a chemiluminescent assay kit (Bio‐rad, USA). The protein expression levels were normalized to β‐actin. Densitometric analysis was conducted with ImageJ software to measure relative protein expression.

#### 2.1.9 Analysis of Gut Microbiota

Following the final administration, the feces of rats in each group were collected in 1.5 mL sterilized EP tubes, frozen in liquid nitrogen, and stored in a −80°C refrigerator for future use. We amplified 16S rRNA's V3‐V4 region for fecal bacteria analysis utilizing primers 341F (5′‐CCTAYGGGRBGCASCAG‐3′) and 806R (5′‐GGACTACNNGGGTATCTAAT‐3′), and Shanghai Meiji Biomedical Technology Co. in China provided sequencing service. Then, we determined flora disparities at genus and phylum levels and analyzed related data on the df online Majorbio Cloud Platform (www.majorbio.com).

### 2.2 Network Pharmacology Analysis

#### 2.2.1 HQGG Potential Active Compounds and Target Acquisition

Details of *Astragali Radix* and *Puerariae Lobatae Radix* were available in Traditional Chinese Medicine Systems Pharmacology (TCMSP), through which the targets of the candidate active compounds in HQGG were screened. Additionally, relevant information, including the name, gene ID, and organism, was obtained from the UniProt Protein Sequence Resource database (http://www.Uniprot.org/). Active compounds without corresponding targets were then removed, and an active compound‐target dataset was created.

#### 2.2.2 Predicting the Targets of MAFLD

In the GeneCards database, using “MAFLD” and “Metabolic related fatty liver disease” as keywords, the MAFLD disease protein targets and their corresponding gene names were searched. Targets with relevance scores ≥ 50 were selected as disease research targets.

#### 2.2.3 Protein–Protein Interaction (PPI) Network Construction and Hub Gene Analysis

Intersection of HQGG bioactive compounds targets and MAFLD‐associated targets was performed utilizing the online website (https://bioinfogp.cnb.csic.es/tools/venny/index.html). Next, the protein–protein interactions of common target genes were analyzed using the STRING database (http://string‐db.org/), and the PPI network was visualized utilizing Cytoscape. Hub genes of HQGG against MAFLD were computed via the Cytohubba (http://apps.cytoscape.org/apps/cytohubba) plugin by the MCC algorithm.

#### 2.2.4 GO and KEGG Enrichment Analysis

The GO and KEGG enrichment analysis for the above obtained targets of HQGG against MAFLD was conducted by DAVID 6.8 (https://david.ncifcrf.gov/) online enrichment analysis platform, while the data were organized and visualized by Bioinformatics (http://www.bioinformatics.com.cn/).

#### 2.2.5 Compound‐Target‐Pathway (C‐T‐P) Network Construction

The “C‐T‐P” network of the active compounds and the key targets was constructed using Cytoscape software. The intersecting targeted genes between MAFLD and the HQGG active compounds were obtained through the pathway from KEGG analysis. In these networks, nodes represent compounds, targets, and pathways, while the connecting lines represent the relationships between biomolecules. We use the Network Analyzer plugin in Cytoscape version 3.7.0 to evaluate the degree, which indicates the number of edges interacting with each node.

#### 2.2.6 Molecular Docking

The top four active compounds of HQGG that act on MAFLD were separately docked with the top five core targets to predict and acquire the binding mode and electrostatic force of the protein–molecule interaction. The 3D structure of the target protein was obtained from the PDB database (https://www.wwpdb.org/) and the HQGG active ingredients mol2 files from the Pubchem database (https://pubchem.ncbi.nlm.nih.gov/), then the molecular docking platform (https://mcule.com) was used for molecular docking. We imported the mol2 files of the HQGG active ingredients and the PDBID of the target protein into the database to obtain 5 docking morphologies, and the conformation with the lowest binding energy was selected as the optimal docking result. Binding energy was employed to assess the binding activity and the docking efficacy of the interactions between the ligand and the protein.

### 2.3 Statistical Analysis

In this research, all measurements were presented as means ± standard deviation and analyzed using one‐way analysis of variance (ANOVA). *p* < 0.05 was statistically significant. All analyses were conducted using SPSS 17.0 statistical software.

## 3 Results

### 3.1 HQGG Alleviates HFD‐Induced Hepatocellular Injury in MAFLD Model Rat

**FIGURE 2:**
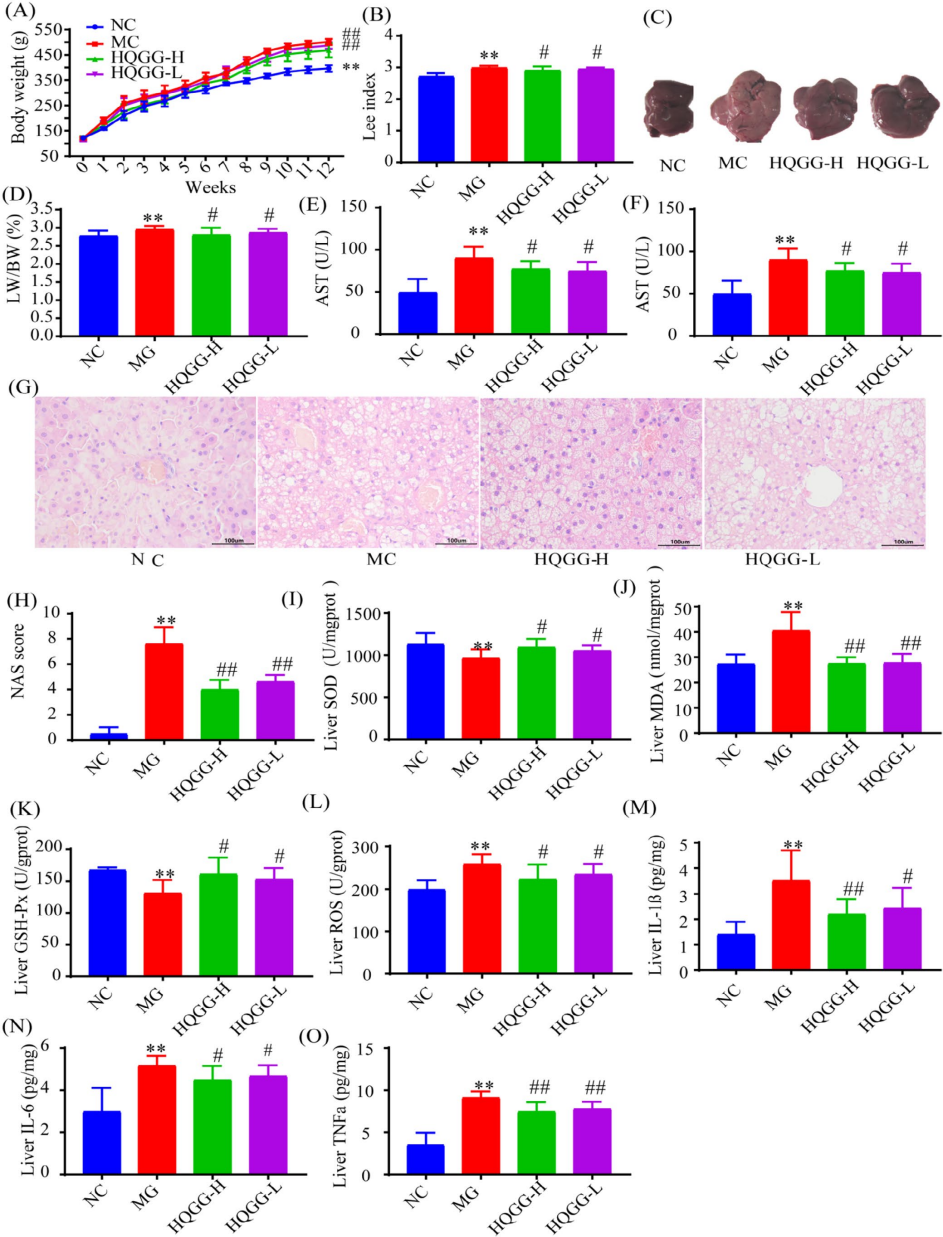
HQGG alleviates HFD‐induced hepatocellular injury in the MAFLD model rat. (A) Body weight of all rats was monitored during the research. Wet weight. (B) Lee index, which is measured using the following formula: [cube root of body weight (g)/naso–anal length (cm) × 1000]. (C) Liver photographs. (D) Liver index. (E) Effects of HQGG on serum AST after treatment. (F) Effects of HQGG on serum ALT after treatment. (G) Representative photomicrographs of HE staining (× 400). (H) The NAS scores. (I) Effects of HQGG on liver SOD after treatment. (J) Effects of HQGG on liver MDA after treatment. (K) Effects of HQGG on liver GSH‐Px after treatment. (L) Effects of HQGG on liver ROS after treatment. (M) Effects of HQGG on liver IL‐1β after treatment. (N) Effects of HQGG on liver IL‐6 after treatment. (O) Effects of HQGG on liver TNF‐α after treatment. Values are shown as mean ± SD. ^#^ *p* < 0.05, ^##^ *p* < 0.01 versus MC and *p < 0.05, **p < 0.01 versus NC.

We established the MAFLD model by feeding HFD to examine the effect of HQGG against MAFLD. As shown in Figure 2, after 12 weeks of administration, MC group rats showed noticeable pathological changes of MAFLD, while body weight (Figure 2A,B) and liver wet weight (Figure 2C,D) of MAFLD rats were significantly reduced in the HQGG treatment group, both HQGG‐L and HQGG‐H groups. Compared with the NC group, the serum ALT and AST activity both greatly improved in HFD‐induced MAFLD rats (*p* < 0.01), while administering the HQGG for 12 weeks, the serum ALT and AST significantly decreased (*p* < 0.05) when comparing with the MC (Figure 2E,F).

The liver histological evaluation revealed that HFD‐induced hepatic steatosis and hepatocyte ballooning in rats, but HQGG (3, 6 g/kg) mitigated these effects (Figure 2G). Simultaneously, the NAS in the MC (Model Control) group was significantly higher than in the NC (Normal Control) group, but it decreased after HQGG (High‐Quality Green Grass) treatment. This indicated that HQGG can significantly reduce liver tissue inflammation, steatosis, and swelling caused by chronic dyslipidemia (Figure 2H).

Oxidative stress is closely related to the development of MAFLD, and nearly all patients with MAFLD exhibit oxidative stress (Yesilova et al. 2005). As shown in Figure 2I–L, compared with NC, the levels of MDA and ROS in the liver were significantly increased in MC rats (*p* < 0.01). After administration of HQGG for 12 weeks, the MDA and ROS in the liver were significantly decreased compared with MC (*p* < 0.05, 0.01). Meanwhile, compared to the NC, the activity of SOD and GSH‐Px in the liver of MC rats was significantly reduced (*p* < 0.01). After 12 weeks of administering HQGG, the activity of SOD and GSH‐Px in the liver was significantly increased compared with the MC (*p* < 0.05).

Inflammation plays a significant role in the progression of MAFLD. Compared with the liver tissue in the NC group, TNF‐α, IL‐1β, and IL‐6 in the liver of the MC were all significantly elevated (*p* < 0.01). Conversely, the levels of hepatic TNF‐α, IL‐1β, and IL‐6 were decreased after HQGG intervention (*p* < 0.05, 0.01) (Figure 2M–O).

Collectively, HQGG can alleviate hepatocellular injury, decrease liver inflammation, and oxidative stress in the HFD‐induced MAFLD model rats.

### 3.2 HQGG Ameliorated Liver Lipid Accumulation in MAFLD Model Rat

**FIGURE 3:**
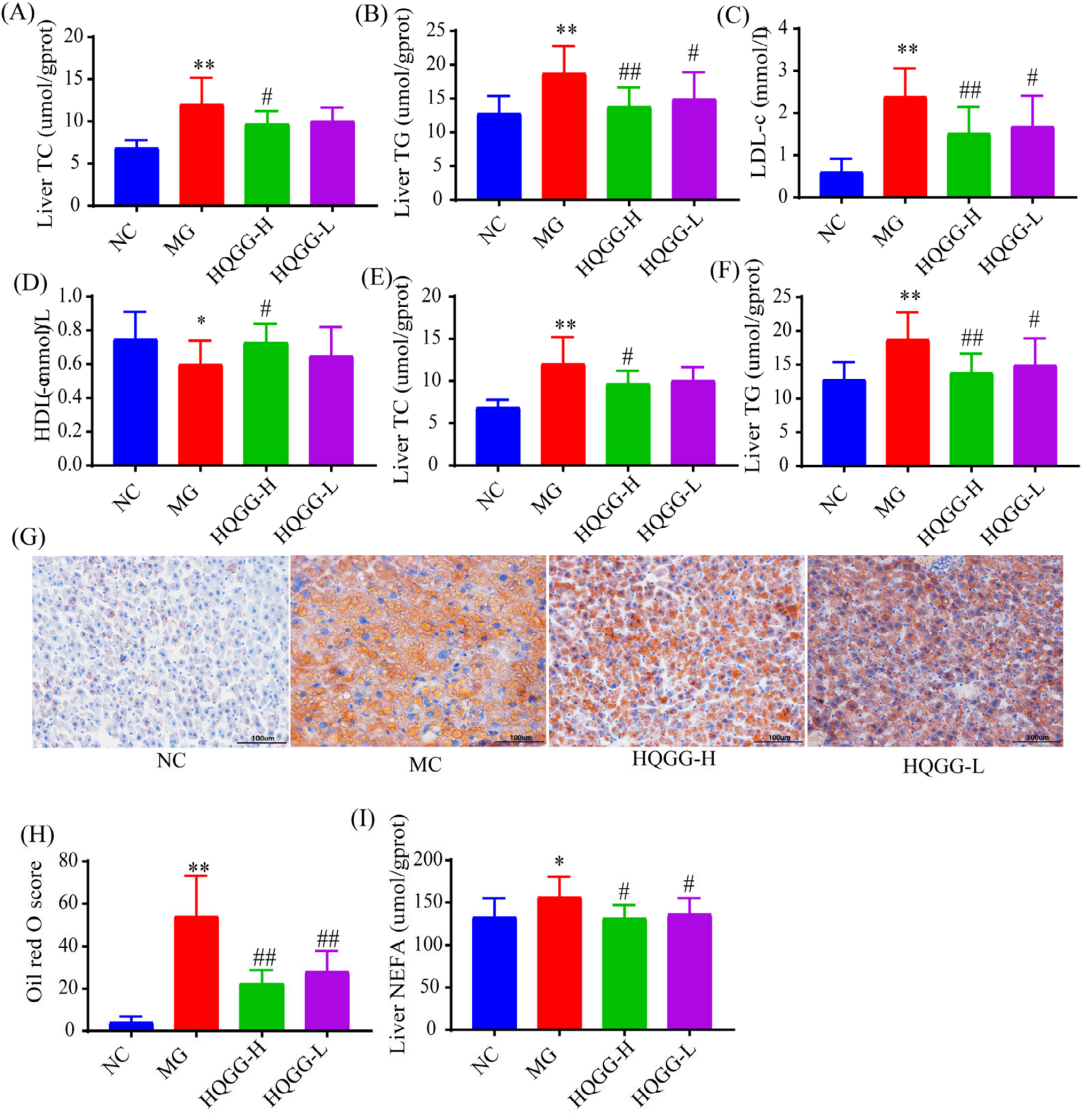
HQGG ameliorated liver lipid accumulation in MAFLD model rats. (A) Effects of HQGG on serum TC after treatment. (B) Effects of HQGG on serum TG after treatment. (C) Effects of HQGG on serum HDL‐c after treatment. (D) Effects of HQGG on serum LDL‐c after treatment. (E) Effects of HQGG on liver TC after treatment. (F) Effects of HQGG on liver TG after treatment. (G) Representative photomicrographs of Oil‐Red O staining (×200). (H) The OD of Oil‐Red O staining (fold change). (I) Effects of HQGG on liver NEFA after treatment. Values are shown as mean ± SD. ^#^ *p* < 0.05, ^##^ *p* < 0.01 versus MC and *p < 0.05, **p < 0.01 versus NC.

Long‐term lipid metabolism disorder can lead to liver lipid damage. As shown in Figure 3, liver and serum lipids were all elevated in the model group. In contrast, after 12 weeks of HQGG treatment, the lipids in the liver and serum were also considerably decreased (*p* < 0.05, 0.01) (Figure 3A,B,D–F,H), and higher HDL‐c levels were observed in the HQGG compared to the MC (Figure 3C).

Oil‐red O staining revealed lipid accumulation in both the MC and HQGG groups compared to the NC. However, the HQGG group exhibited less lipid accumulation than the MC (Figure 3G,I).

### 3.3 Network Pharmacology Analysis

To further elucidate the mechanism of HQGG in treating MAFLD, we conducted a preliminary exploration by network pharmacology.

#### 3.3.1 HQGG Potential Active Compounds and Targets

The active ingredients in HQGG (*Astragali Radix* and *Puerariae Lobatae Radix*) were retrieved via the TCMSP database. Subsequently, a total of 24 active ingredients were screened according to the ADME model (OB ≥ 30% and DL ≥ 0.18). The specific information of active ingredients is shown in Table 1.

**TABLE 1:** Chemical information of 24 active compounds in HQGG.

Herbal medicine	Mol ID	Molecule name	MW	OB (%)	DL
*Pueraria lobata*	MOL000392	Formononetin	268.28	45.97	0.19
	MOL000358	Beta‐Sitosterol	414.79	42.1	0.2
	MOL002959	3′‐Methoxydaidzein	284.28	34.97	0.24
	MOL003629	Daidzein‐4,7‐diglucoside	578.57	39.84	0.71
	MOL012297	Puerarin	416.41	56.45	0.39
Radix Astragali	MOL000211	Mairin	456.78	55.38	0.78
	MOL000239	Jaranol	314.31	50.83	0.29
	MOL000296	Hederagenin	414.79	36.91	0.75
	MOL000033	(3S,8S,9S,10R,13R,14S,17R)‐10,13‐dimethyl‐17‐[(2R,5S)‐5‐propan‐2‐yloctan‐2‐yl]‐2,3,4,7,8,9,11,12,14,15,16,17‐dodecahydro‐1H‐cyclopenta[a]phenanthren‐3‐ol	428.82	36.23	0.78
	MOL000354	Isorhamnetin	316.28	49.6	0.31
	MOL000371	3,9‐di‐O‐methylnissolin	314.36	53.74	0.48
	MOL000374	5′‐hydroxyiso‐muronulatol‐2′,5′‐di‐O‐glucoside	642.67	41.72	0.69
	MOL000378	7‐O‐methylisomucronulatol	316.38	74.69	0.3
	MOL000379	9,10‐dimethoxypterocarpan‐3‐O‐β‐D‐glucoside	462.49	36.74	0.92
	MOL000380	(6aR,11aR)‐9,10‐dimethoxy‐6a,11a‐dihydro‐6H‐benzofurano[3,2‐c]chromen‐3‐ol	300.33	64.26	0.42
	MOL000387	Bifendate	418.38	31.1	0.67
	MOL000392	Formononetin	268.28	69.67	0.21
	MOL000398	Isoflavanone	316.33	109.99	0.3
	MOL000417	Calycosin	284.28	47.75	0.24
	MOL000422	Kaempferol	286.25	41.88	0.24
	MOL000433	FA	441.45	68.96	0.71
	MOL000438	(3R)‐3‐(2‐hydroxy‐3,4‐dimethoxyphenyl) chroman‐7‐ol	302.35	67.67	0.26
	MOL000439	Isomucronulatol‐7,2′‐Di‐O‐Glucosiole	626.67	49.28	0.62
	MOL000442	1,7‐Dihydroxy‐3,9‐dimethoxy pterocarpene	314.31	39.05	0.48
	MOL000098	Quercetin	302.25	46.43	0.28

The candidate active compounds of HQGG were screened through the TCMSP database to identify their targets. Following the removal of active compounds without identifiable targets, 21 active compounds and 238 corresponding targets were obtained.

#### 3.3.2 Compound‐Target (C‐T) Network Analysis

**FIGURE 4:**
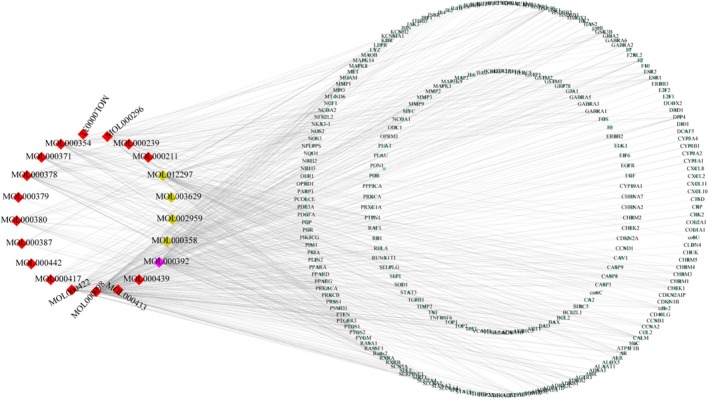
Compound‐target network. There were 259 nodes (21 bioactive compound nodes, 238 target nodes) and 614 edges in this network, and the red node represents the compounds, and the green refers to the targets.

The HQGG Active Compound‐Target (C‐T) network was constructed using the Cytoscape 3.7.0 platform (see Figure 4). There are 259 nodes (21 bioactive ingredients, 238 targets) and 614 edges in the network. Green nodes represent HQGG active ingredients, while red nodes signify action targets. Quercetin (MOL000098, degree = 154), formononetin (MOL000392, degree = 78), kaempferol (MOL000422, degree = 63), and puerarin (MOL012297, degree = 55) have higher degree values and stronger interactions with the targets, which may be the core active ingredients of HQGG. In addition, candidate targets of PTGS2 (degree = 17), PTGS1 (degree = 14), PPARG (degree = 13), HSP 90 (degree = 13), and PRSS1 (degree = 12) may be the primary action targets of their active ingredients.

#### 3.3.3 Integration of the PPI Network and Analysis of Hub Gene

**FIGURE 5:**
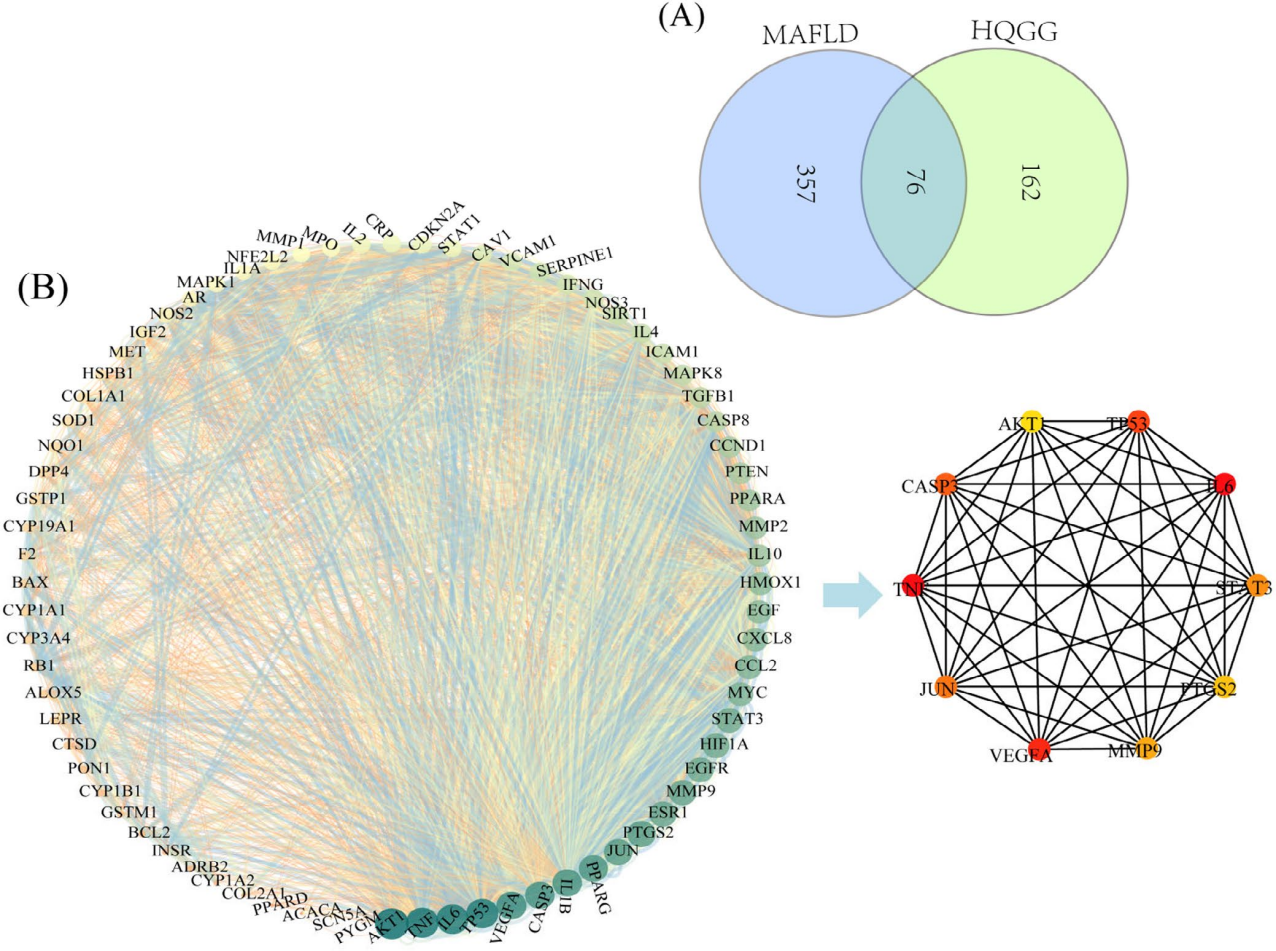
Venn diagram of targets and PPI network of HQGG treating MAFLD. (A) Venn diagram was set up by an online website to acquire the 76 common targets between the HQGG bioactive ingredients targets and the MAFLD‐associated targets. (B) These target genes were inputted into the STRING online website, and the PPI network made up of 76 inter‐action nodes and 1373 interaction edges. Nodes refer to core target genes. The size of the nodes and edges matches the value of degree and integrate mark respectively. The color of the nodes refers to the value of degree. The darker the color (green), the higher the degree. (C) Hub gene of HQGG against MAFLD was calculated by Cytohubba plugin by MCC algorithm, the 10 nodes with the largest degree value were chosen as the hub genes.

A total of 433 MAFLD‐related targets were identified from the GeneCards database. These were matched with 238 targets of active compounds in HQGG, resulting in 76 targets of active compounds in HQGG that act on MAFLD, as shown in Table 2. Using Venny2.1.0 (https://bioinfogp.cnb.csic.es/tools/venny/index.html) online mapping tools to get drug‐disease targets Venn diagram (as shown in Figure 5A). Within the network visualization, blue vertices correspond to drug targets, yellow vertices denote disease targets, while gray vertices illustrate shared components within the drug‐disease interactome.

**TABLE 2:** Target information of the active ingredients in HQGG against MAFLD.

Disease	Targets number	Targets
MAFLD	76	NOS2, ESR1, AR, PPARG, PTGS2, ADRB2, DPP4, F2, NOS3, JUN, IL4, SIRT1, SCN5A, BCL2, BAX, CASP3, CASP8, TGFB1, PON1, STAT3, AKT1, VEGFA, MMP2, MMP9, TNF, SOD1, HIF1A, VCAM1, CYP19A1, GSTP1, LEPR, PYGM, PPARD, MET, MAPK8, MMP1, STAT1, HMOX1, CYP3A4, CYP1A2, CYP1A1, ICAM1, CYP1B1, ALOX5, INSR, GSTM1, EGFR, CCND1, MAPK1, IL10, EGF, RB1, IL6, CDKN2A, TP53, ACACA, CAV1, MYC, IL1B, CCL2, CXCL8, HSPB1, IL2, SERPINE1, COL1A1, IFNG, PTEN, IL1A, MPO, NFE2L2, NQO1, COL2A1, PPARA, CRP, CTSD, IGF2

The 76 common targets of HQGG bioactive compounds and MAFLD were input into the STRING website (PPI score > 0.4), resulting in a PPI network composed of 76 interaction nodes and 1373 interaction edges. As depicted in Figure 5B, the chromatic intensity of nodes demonstrates a positive correlation with their connection density, where deeper crimson hues signify heightened nodal centrality within the network architecture.

Utilizing the Cytoscape plugin Cytohubba, hub genes were meticulously screened within the interaction network. Specifically, the MCC (Maximal Clique Centrality) algorithm was applied to pinpoint the top 10 hub genes in the context of HQGG treatment for MAFLD (Figure 5C), which were PTGS2, JUN, VEGFA, STAT3, MMP9, AKT1, IL6, TP53, TNF, and CASP3.

#### 3.3.4 GO Functional Enrichment and KEGG Pathway Analysis

**FIGURE 6:**
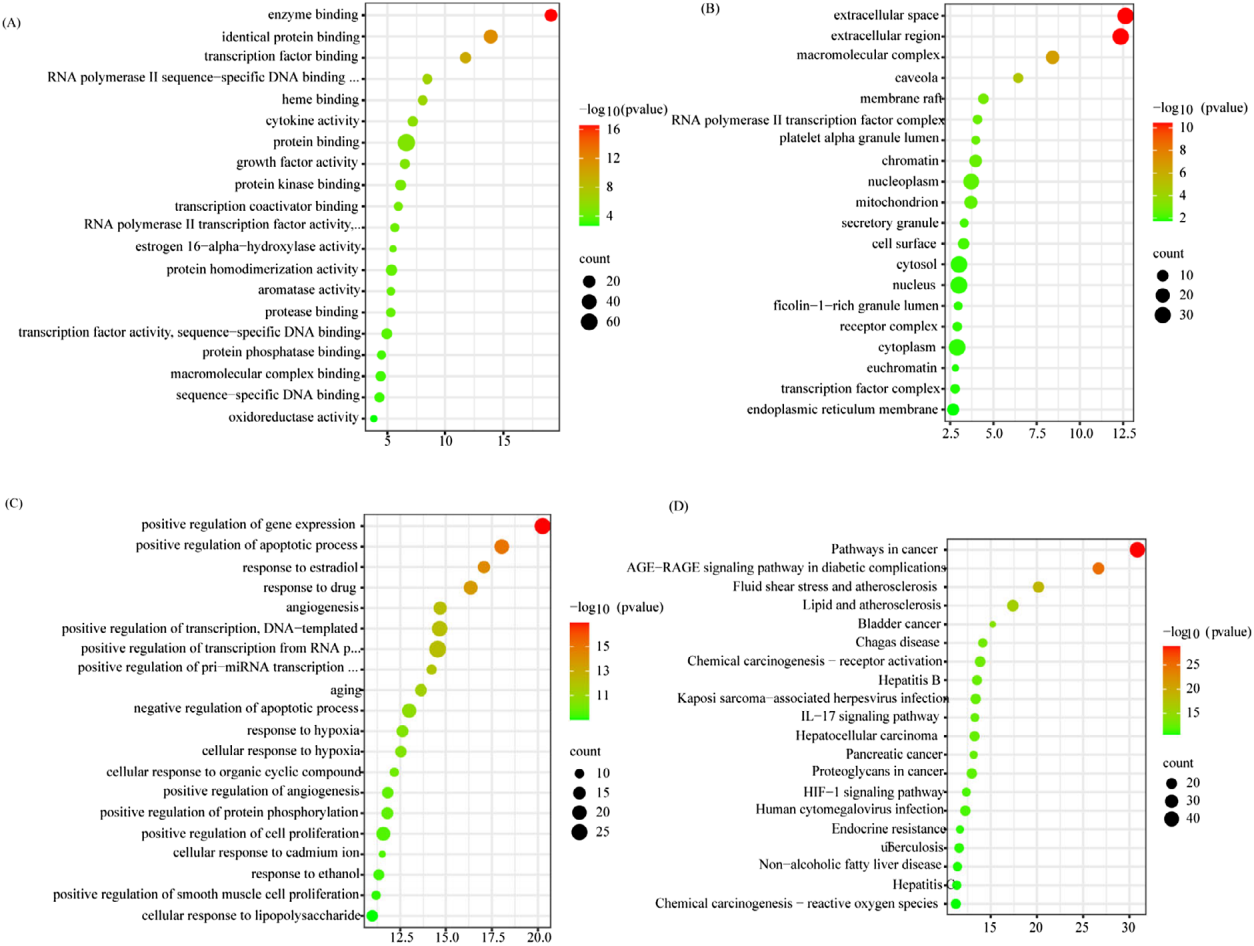
GO functional enrichment and KEGG pathway analysis with DAVID database. (A) The biological processes enrichment analysis. (B) The molecular functions enrichment analysis. (C) The cellular component enrichment analysis. (D) The KEGG pathway analysis. Cross‐target genes related to MAFLD and the HQGG active compounds associated as bits were used to obtain corresponding functions from DAVID, and then import target genes into the DAVID database for GO analysis, biological process, and KEGG pathway analysis. The Y‐axis represents the class of greatly enhanced biological process categories associated with target genes, and the X‐axis represents the log10 (*P* value). The number of target genes in the pathway is represented by the size of the dots, and the difference in the FDR scope is represented by the color of the dots.

To elucidate the mechanism of HQGG in treating MAFLD at an integrative level, GO enrichment analysis was conducted on the biological processes, molecular functions, and cellular components of 76 shared targets. Figure 6A–D lists the top 10 significantly GO enriched for these targets (FDR < 0.05). The results showed that the targets of HQGG were closely associated with five biological processes: positive regulation of gene expression, positive regulation of apoptotic process, response to estradiol, response to drug, and angiogenesis; five molecular functions: extracellular space, extracellular region, macromolecular complex, caveola, and membrane raft; five cell components: enzyme binding, identical protein binding, transcription factor binding, RNA polymerase II sequence‐specific DNA binding, transcription factor binding, and heme binding.

As shown in Figure 6D, the top 20 significantly enriched KEGG pathways of these targets were analyzed (FDR < 0.05). The results indicate that these targets are primarily associated with several signaling pathways, including the AGE‐RAGE signaling pathway in diabetic complications, fluid shear stress and atherosclerosis, lipid metabolism and atherosclerosis, IL‐17 signaling pathway, non‐alcoholic fatty liver disease, and so on (see Table 3).

**TABLE 3:** Based on KEGG enrichment and C‐T‐P network analysis, we picked out 5 important signaling pathways that were significantly associated with HQGG treatment of MAFLD.

Term	ID	Input number	*p*	Input gene name
AGE‐RAGE signaling pathway in diabetic complications	hsa04933	24	2.22E‐27	JUN, TGFB1, VCAM1, CXCL8, NOS3, STAT1, MMP2, STAT3, SERPINE1, TNF, ICAM1, VEGFA, COL1A1, IL1A, IL6, MAPK8, CCND1, IL1B, CASP3, BCL2, BAX, CCL2, AKT1, MAPK1
Lipid and atherosclerosis	hsa05417	23	4.09E‐18	JUN, VCAM1, CXCL8, NOS3, MMP1, STAT3, TNF, MMP9, ICAM1, IL6, MAPK8, CASP8, IL1B, CASP3, CYP1A1, BCL2, BAX, CCL2, AKT1, MAPK1, PPARG, TP53, NFE2L2
Fluid shear stress and atherosclerosis	hsa05418	22	6.82E‐21	NQO1, JUN, GSTM1, VCAM1, NOS3, GSTP1, MMP2, CAV1, TNF, MMP9, ICAM1, VEGFA, IL1A, MAPK8, IFNG, IL1B, BCL2, CCL2, AKT1, HMOX1, TP53, NFE2L2
Non‐alcoholic fatty liver disease	hsa04932	16	3.99E‐12	JUN, TGFB1, CXCL8, INSR, TNF, IL1A, IL6, MAPK8, CASP8, IL1B, CASP3, LEPR, BAX, AKT1, PPARG, PPARA
IL‐17 signaling pathway	hsa04657	15	5.27E‐14	JUN, CXCL8, MMP1, PTGS2, TNF, MMP9, IL4, IL6, MAPK8, CASP8, IFNG, IL1B, CASP3, CCL2, MAPK1

#### 3.3.5 Compound‐Target‐Pathway for HQGG Against MAFLD Network Analysis

**FIGURE 7:**
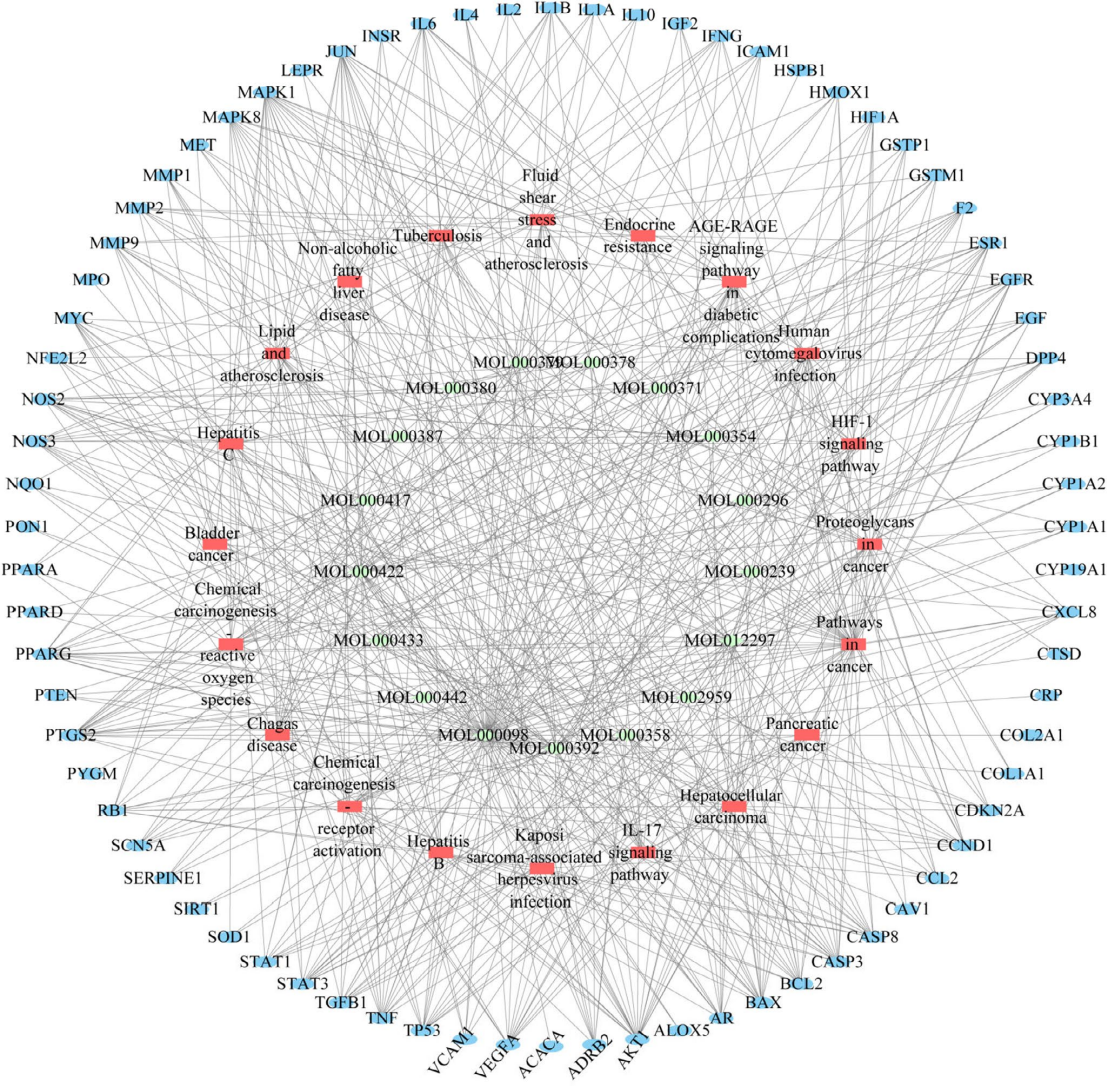
Compounds‐targets‐pathways for HQGG against MAFLD network analysis. The core pathways obtained by C‐T‐P network analysis are assembled (green nodes are compounds, red nodes are pathways, and blue nodes are targets).

To construct the “complex targeting pathway (C‐T‐P)” network as shown in Figure 7, C‐T‐P network analysis found that quercetin (MOL000098, degree = 68), kaempferol (MOL000422, degree = 28), formononetin (MOL000392, degree = 26), and puerarin (MOL012297, degree = 23) had high degree values, suggesting that these substances may be key active ingredients in anti‐MAFLD.

By analyzing the C‐T‐P network, five key targets for HQGG treatment of MAFLD and five significant signaling pathways were identified. The top five targets are PTGS2 (degree = 21), AKT1 (degree = 21), MAPK1 (degree = 19), JUN (degree = 18), and PPARG (degree = 16), which may be the primary targets of HQGG against MAFLD. The five chosen pathways include the AGE‐RAGE signaling pathway in diabetic complications (degree = 24), lipid and atherosclerosis (degree = 23), fluid shear stress and atherosclerosis (degree = 22), non‐alcoholic fatty liver disease (degree = 16), and the IL‐17 signaling pathway (degree = 15).

#### 3.3.6 Molecular Docking Result

**FIGURE 8:**
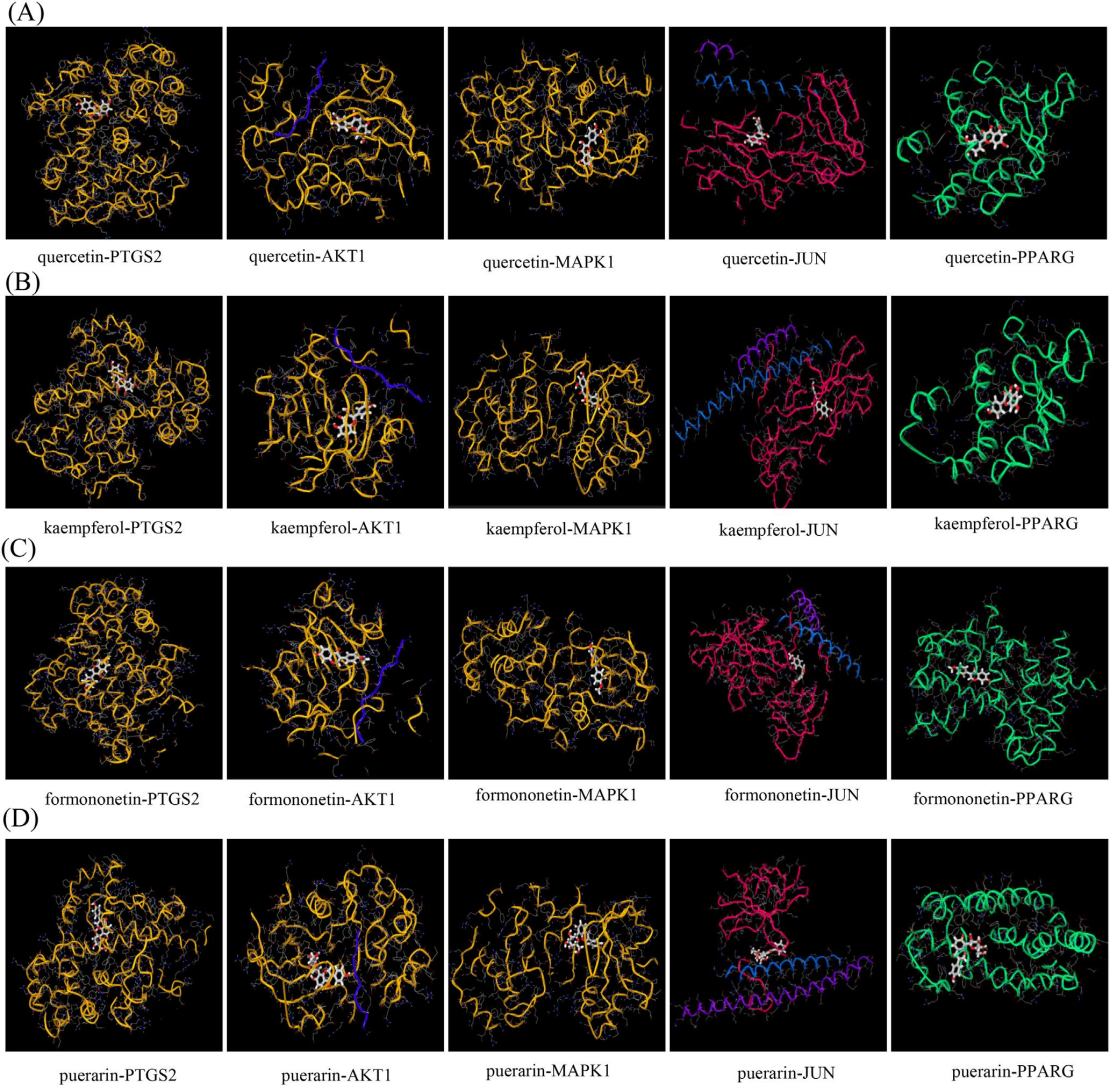
Docking pattern diagram of active components and key target molecules in HQGG. (A) Quercetin interacts with key targets PTGS2, AKT1, MAPK1, JUN, and PPARG, respectively. (B) Kaempferol interacts with key targets PTGS2, AKT1, MAPK1, JUN, and PPARG, respectively. (C) Formononetin interacts with key targets PTGS2, AKT1, MAPK1, JUN, and PPARG, respectively. (D) Puerarin interacts with key targets PTGS2, AKT1, MAPK1, JUN, and PPARG, respectively.

By integrating “PPI” and “C‐T‐P” network analysis, the core active ingredients of HQGG, including quercetin, kaempferol, formononetin, and puerarin, were predicted to bind to key targets PTGS2, AKT1, MAPK1, JUN, and PPARG. It is generally believed that a binding energy below −4.25 kcal·mol^−1^ indicates that the ligand and the receptor have a certain binding activity, a binding energy under −5.0 kcal·mol^−1^ indicates a better binding activity, while less than −7.0 kcal·mol^−1^ has vigorous binding activity (Hsin et al. 2013). Pairing four active ingredients with five target proteins using the mcule Molecular docking platform (https://mcule.com), the results showed that quercetin, kaempferol, formononetin, and puerarin had good binding ability with PTGS2, AKT1, MAPK1, JUN, and PPARG (Table 4, Figure 8).

**TABLE 4:** Forecasting the binding energy between active compounds and primary targets in HQGG.

Compounds	PTGS2	AKT1	MAPK1	JUN	PPARG
Quercetin	−8.4	−9.1	−8.5	−6.1	−7.6
Kaempferol	−8.5	−8.8	−7.9	−6.0	−7.6
Formononetin	−8.5	−8.4	−8.2	−5.7	−8.0
Puerarin	−7.4	−8.5	−8.8	−7.0	−5.2

### 3.4 Molecular Verification of MAFLD Modified by HQGG Therapy

To further elucidate how HQGG affects MAFLD, the protein levels of the primary target and the PPAR‐γ/NF‐κB signaling pathway, as predicted by network pharmacology, were confirmed using RT‐PCR and western blot analysis.

**FIGURE 9:**
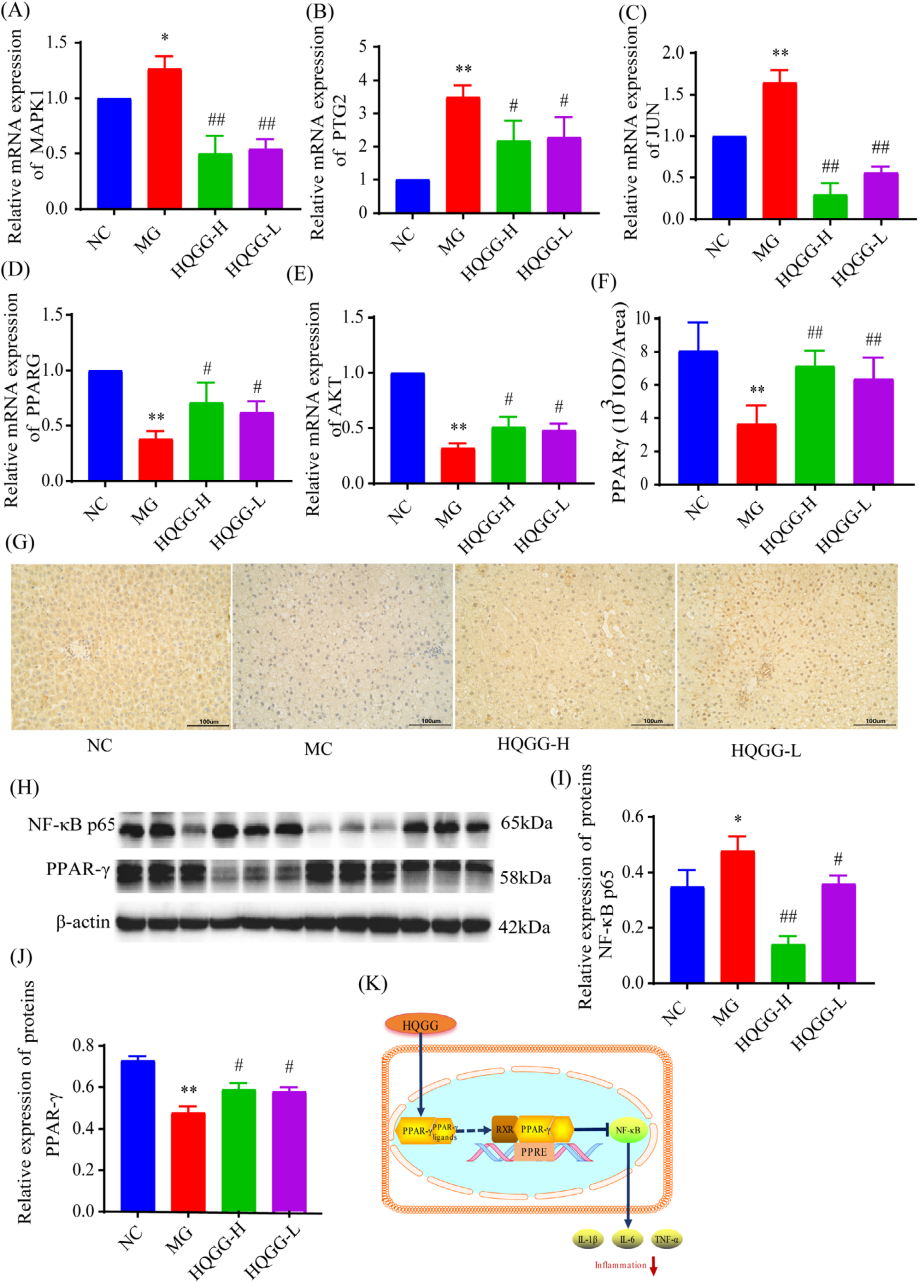
Molecular verification of MAFLD modified by HQGG therapy. (A) The mRNA expressions of MAPK1 in the liver. (B) The mRNA expressions of PTGS2 in the liver. (C) The mRNA expressions of *JUN* in the liver. (D) The mRNA expressions of *PPARG* in the liver. (E) The mRNA expressions of *AKT1* in the liver. (F) Relative optical density of liver PPAR‐γ. (G) Representative photomicrograph of liver PPAR‐γ protein expression by IHC (×400). (H) Effects of HQGG on expression levels of PPAR‐γ and NF‐κB in MAFLD rats. (I) Statistical map of NF‐κB protein expression in liver tissue. (J) Statistical map of PPAR‐γ protein expression in liver tissue. (K) The molecular verification mechanism diagram of HQGG therapy for MAFLD. Values are shown as mean ± SD. ^#^ *p* < 0.05, ^##^ *p* < 0.01 versus MC and *p < 0.05, **p < 0.01 versus NC.

As displayed in Figure 9A–E, the mRNA expressions of PTGS2, MAPK1, and JUN in the liver of the MC were higher than in the NC (*p* < 0.05), while AKT1 and PPARG were significantly decreased compared to the NC (*p* < 0.05). Compared with the MC, transcription levels of AKT1 and PPARG were greatly increased by HQGG therapy (*p* < 0.05), while there were significant decreases in PTGS2, MAPK1, and JUN at mRNA levels (*p* < 0.05).

In this study, immunochemical staining was used to stain PPAR‐γ in liver tissue. Compared to the NC, the liver of the MC showed a significant reduction in PPAR‐γ expression. However, HQGG treatment reversed this trend (*p* < 0.01) (Figure 9F,G). This aligns with the prediction that PPARG is a key player in the PPI network of HQGG in MAFLD.

Based on the outcomes of network pharmacology and experimental validation, the PPAR‐γ signaling pathway emerged as the most relevant pathway associated with MAFLD. Consequently, the hub targets of this pathway, namely PPAR‐γ and NF‐κB, which are integral to the PPAR‐γ signaling pathway, were selected for further exploration of their potential mechanisms in this study. As expected, the levels of NF‐κB were considerably increased, and PPAR‐γ significantly decreased after feeding a high‐fat diet. However, their expressions of NF‐κB were significantly downregulated, and PPAR‐γ considerably increased after HQGG treatment (Figure 9H–K).

### 3.5 Impacts of HQGG on Gut Microbiota in MAFLD Rats

**FIGURE 10:**
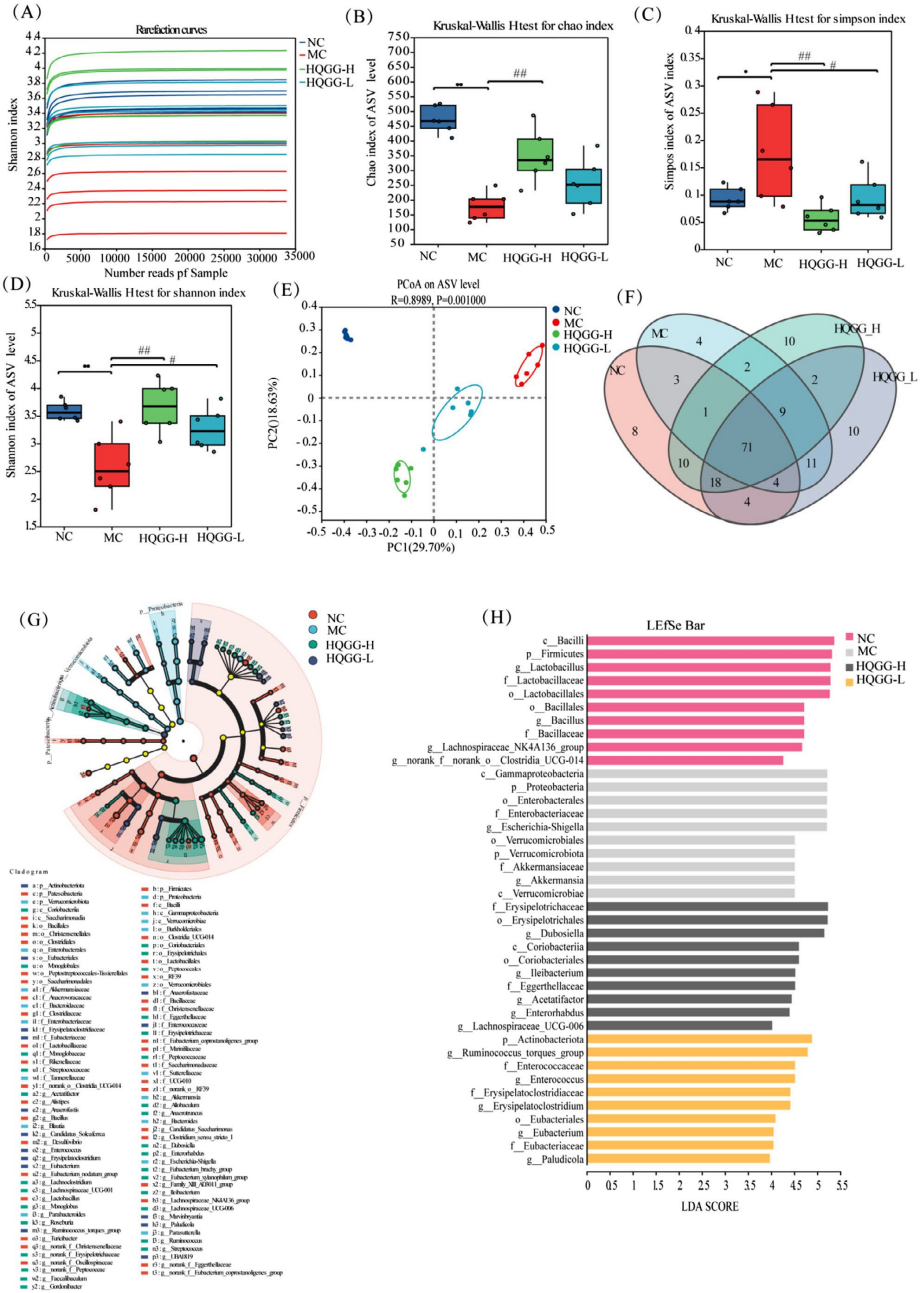
Impacts of HQGG upon gut microbiota in MAFLD rats. (A) Shannon index curve. (B) Chao indexes. (C) Shannon indexes. (D) Simpson indexes. (E) Gut microbiota's 3D principal coordinate analysis (PCoA) maps in mice, founded upon weighted Unifrac method. (F) Venn diagram analysis. (G) Cladogram exhibiting LEfSe analysis outcomes. (H) LDA scores denote the bacterial taxa enrichment level in gut microbiota. Values are denoted by mean ± SD. ^#^ *p* < 0.05, ^##^ *p* < 0.01 versus MG and *p < 0.05, **p < 0.01 versus NG.

The gut microbiota is considered to play a causal role in the pathogenesis of MAFLD (Schwenger et al. 2019). To assess the effect of HQGG on the structural variations of gut microbiota, 16S rRNA gene sequences were analyzed from fecal specimens. Observed from Figure 10A, The Shannon rarefaction curve shows that rank abundance declined smoothly and eventually flattened, demonstrating that diversity levels had been up to confidence interval. The α‐diversity analysis revealed that, compared to the NC, the Chao index and Shannon index in the MC were significantly lower, while the Simpson index was significantly increased; In contrast, the HQGG groups increased dramatically regarding α‐diversity indices in comparison with MC, indicative of gut microbiota diversity (Figure 10B–D).

Principal coordinate analysis (PCoA) using UniFrac distances showed distinct clustering patterns among gut microbial communities across various test groups. MG and HQGG treatments cause significant changes in microbial community structure (Figure 10E). A Venn diagram depicted 167 operational taxonomic units (OTUs) totally identified across all specimens, with 71 OTUs shared among disparate test groups. The NC, HQGG‐H, and HQGG‐L groups demonstrated 119, 129, and 123 distinct OTUs, respectively. Conversely, MG showed less OTUs (*n* = 105). Such outcomes mean that HQGG supplementation may recover gut microbiota diversity in MAFLD rats (Figure 10F). Figure 10G,H depict gut microbial taxa's LEfSe analysis outcomes. NC demonstrated enhanced *c_Bacilli, p_Firmicutes*, and *g_Lactobacillus* prevalence. By contrast, MC demonstrated elevated *c_Gammaproteobacteria, p_Proteobacteria*, and *o_Enterobacterales* prevalence. In comparison, HQGG‐H intervention dramatically elevated *f_Erysipelotrichaceae*, *o_Erysipelotrichales*, and *g_Dubosiella* at genus level were found, whereas HQGG‐L upward trend *p_Actinobacteriota*, *g_Ruminococcus_torques_group*, and *f_Enterococcaceae* abundances. Those results indicated that HQGG had a beneficial effect on improving the species richness and diversity of the gut microbiota in MAFLD rats.

**FIGURE 11:**
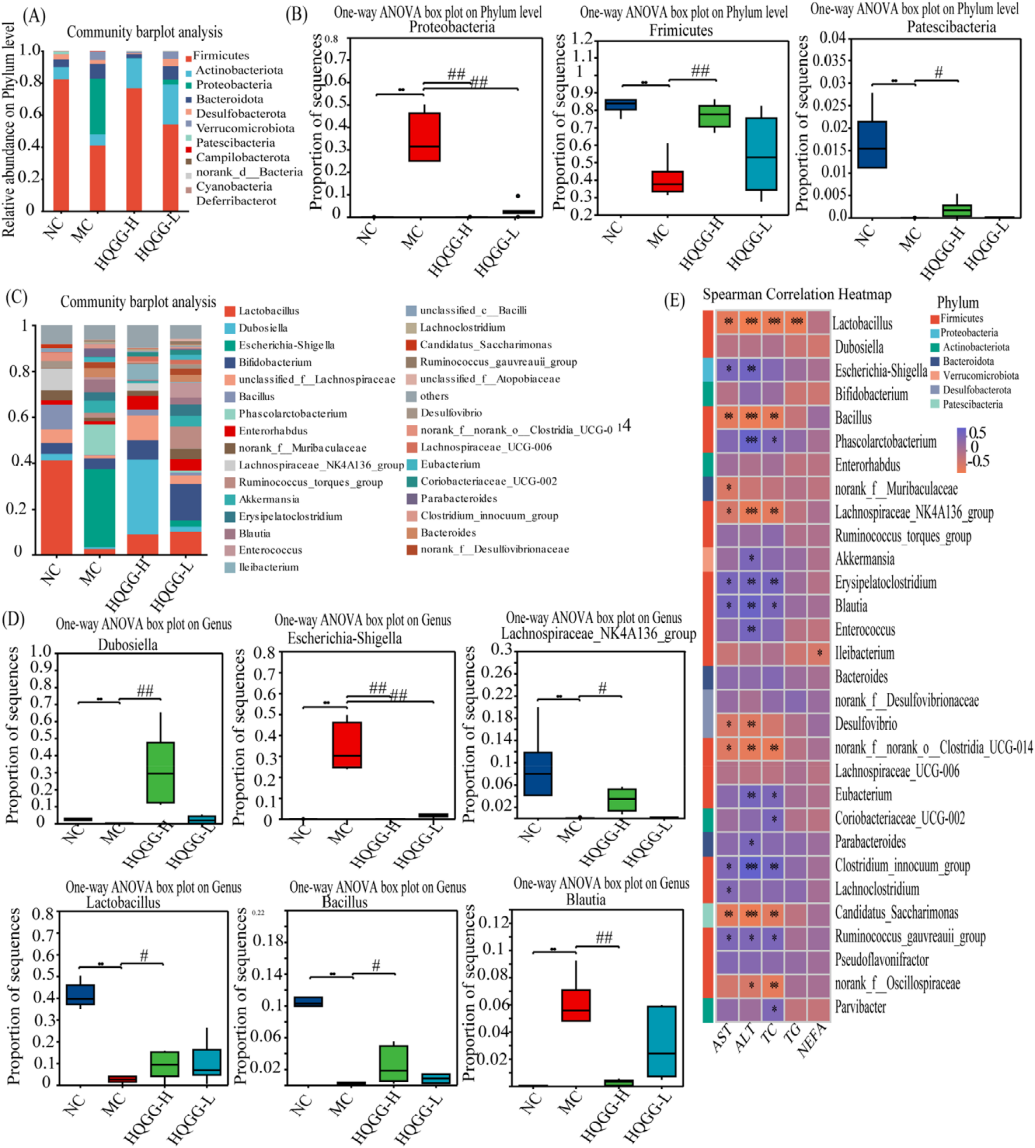
Impacts of HQGG upon gut microbiota structure in MAFLD rats. (A) Gut microbiota composition in fecal specimens at phylum level. (B) Microbiota phylum‐level distribution regarding relative abundance. (C) Gut microbiota composition in fecal specimens at genus level. (D) Microbiota genus‐level distribution regarding relative abundance. (E) Correlation analysis of MAFLD‐related indicators and gut microbiota. Values are denoted by mean ± SD. ^#^ *p* < 0.05, ^##^ *p* < 0.01 versus MG and *p < 0.05, **p < 0.01 versus NG.

The structure of gut microbiota was assessed by taxonomy. The results at the phylum level indicate that the HQGG‐H intervention may increase the population of *Firmicutes* (Figure 11A). Compared with MG, the abundance of *Firmicutes* and *Patescibacteria* was apparently elevated in the HQGG‐H group, the abundance of *Proteobacteria* was significantly reduced in HQGG groups (Figure 11B). At the genus level, the results observed that HQGG intervention dramatically increased relative *Lactobacillus, Bacillus, Lachnospiraceae_NK4A136_group*, and *Dubosiella* abundance to a substantially compared with the MC, and HQGG intervention caused extremely significant reductions in *Escherichia‐Shigella* and *Blautia* compared to the MG (Figure 11C,D).

In order to gain a deeper comprehension of the relationship between alterations in key gut microbiota and MAFLD, the Spearman correlation analysis was conducted. As shown in Figure 11E, at the genus level, out of the top 30 genera affected by HQGG intervention, 22 of them showed a significant negative or positive correlation with at least one indicator (*p* < 0.01). The *Lactobacillus, Bacillus, Lachnospiraceae_NK4A136_group, Candidatus_Saccharimonas*, and *Desulfovibrio* showed a negative association with MAFLD‐related indicators (AST, ALT, and TC). On the other hand, *Escherichia‐Shigella, Phascolarctobacterium, Erysipelatoclostridium, Blautia, Eubacterium*, and *Clostridium_innocuum_group* demonstrated a positive relation with MAFLD‐related indicators.

## 4 Discussion

MAFLD, a global threat to public health, is related to metabolic syndrome, obesity, diabetes, and hyperlipidemia (Targher et al. 2021). In this study, we constructed the MAFLD rat model by feeding HFD to investigate the mechanism of HQGG. The results show that HQGG can alleviate hepatocellular injury, decrease liver inflammation, and oxidative stress in the HFD‐induced MAFLD model rats. According to the knowledge that TCM exerts effects on multiple targets, we applied network pharmacology and conducted animal experiments to explore the potential active ingredients and mechanism of HQGG that function to alleviate MAFLD. This study characterized 21 principal bioactive constituents and 238 corresponding targets in HQGG, revealing 76 targets exhibiting functional overlap with MAFLD pathophysiology. The network analysis revealed that the primary active components of HQGG (quercetin, kaempferol, formononetin, puerarin, etc.) might regulate PTGS2, AKT1, MAPK1, JUN, PPARG, and other targets, and also act on various signaling pathways such as those related to the AGE‐RAGE signaling pathway in diabetic complications, lipid and atherosclerosis, fluid shear stress and atherosclerosis, NAFLD, and the IL‐17 signaling pathway, thereby playing an important role in treating MAFLD. The molecular docking results indicated that the selected HQGG core active compounds had good binding activity with key targets.

Modern pharmacological studies have found that the active ingredient quercetin has a strong anti‐oxidative stress effect and an inhibitory effect on hepatocyte apoptosis, inflammation, and production of reactive oxygen species, which are all factors related to the occurrence of MAFLD (Sotiropoulou et al. 2021). In vitro experiments have shown that kaempferol can regulate lipid metabolism, ameliorate apoptosis, and modulate inflammation and autophagy in the fatty liver cell model (Zhou, Zhang, Bao, and Li 2022). Research has shown that formononetin can significantly alleviate liver steatosis in HFD mice. Formononetin has also been shown to reduce lipid accumulation in HepG2 cells and mouse primary hepatocytes stimulated by FFAs (Wang, Zhao, et al. 2019). Quercetin and kaempferol have been found to effectively inhibit PPAR‐γ activation, thus regulating lipid metabolism in the liver (Mi 2019; Park et al. 2022). Meanwhile, research has confirmed that puerarin could ameliorate high‐fat and high‐fructose diet (HFFD)‐induced MAFLD by modulating liver lipid accumulation, oxidative stress, liver function, and inflammation (Wang, Yang, et al. 2019; Zhou, Zhang, Aldhahrani, et al. 2022). These studies demonstrate the effectiveness of the active ingredients in HQGG for treating MAFLD.

Meanwhile, the network analysis results showed that the treatment of MAFLD by HQGG was highly enriched in the response to lipids and inflammation. The in vivo experiment results confirmed that HQGG ameliorates liver inflammation by inhibiting the release of IL‐1β, IL‐6, and TNF‐α in the liver of MAFLD model rats. A clinical sign of MAFLD is the presence of steatosis in more than 5% of hepatocytes (Eslam and George 2021). Excessive lipid accumulation in the liver serves as a precursor for steatosis, which induces the production of lipid peroxidation and causes lipid toxicity in hepatocytes (Mota et al. 2016). In this study, the results of the hepatic TG, NEFA, liver Oil Red O, and H&E staining evaluations demonstrated that HQGG reduced lipid accumulation in hepatocytes in rats on HFD. Meanwhile, oxidative stress is closely linked to the development of MAFLD, and nearly all MAFLD patients experience oxidative stress (Reis‐Barbosa et al. 2022; Yesilova et al. 2005). In this study, HQGG was found to attenuate liver oxidative stress injury in HFD‐induced rats. These findings indicate that HQGG may reduce liver inflammation in HFD‐induced rats, potentially benefiting MAFLD.

PPARG, JUN, PTGS2, and AKT1 play a crucial role in regulating lipid metabolism, oxidative stress response, and inflammation, and exhibit close interactions among their targets (Chen et al. 2023; Min et al. 2022). Among them, the ligand‐activated transcription factor PPAR‐γ of the nuclear hormone receptor superfamily is considered to be a crucial metabolic regulator of adipogenesis, inflammation, insulin resistance, endoplasmic reticulum stress, oxidative stress, and fibrosis in the pathogenesis of MAFLD (Chen et al. 2023), and PPAR‐γ has also been reported to be strongly induced in preclinical models of MAFLD as well as in the liver of patients (Hasenfuss et al. 2014; Platko et al. 2019).

Hepatic inflammatory responses serve as a central driver in MAFLD progression, wherein activated Kupffer cells secrete pro‐inflammatory mediators, thereby amplifying inflammatory signaling cascades that exacerbate disease pathogenesis. PPAR‐γ has been established as a critical modulator of hepatic lipid homeostasis and inflammatory regulation (Chen et al. 2023). PPAR‐γ modulates inflammatory responses through transcriptional interference with NF‐κB signaling, where its activation downregulates pro‐inflammatory cytokine production, establishing the PPAR‐γ/NF‐κB axis as a key therapeutic target for hepatic inflammatory modulation (Linard et al. 2008). This study revealed that following HQGG intervention, the expression of PPAR‐γ protein markedly increased, the nuclear content of NF‐κB protein significantly decreased, and serum levels of IL‐1β, IL‐6, and TNF‐α were substantially reduced. These results suggest that the mechanism by which HQGG treats MAFLD may be through activating PPAR‐γ, thereby inhibiting the activity of transcription factors such as NF‐κB, and exerting a negative indirect transcriptional regulatory effect, significantly inhibiting the inflammatory response and alleviating liver inflammation in MAFLD rats (Figure 9K).

Gut microbiota significantly influences the development of MAFLD (Li and Hu 2020). TMC not only directly act on the target organs of diseases, but also target the gut microbiota to regulate the types, quantities, and metabolites of the gut microbiota, thereby exerting regulatory effects on host organ functions (Yue et al. 2022). The results of the microbial diversity analysis conducted in this study indicated that HQGG can enhance microbial diversity, increase species richness, and influence the microbial structure of MAFLD rats. At the phylum level, HQGG increased *Firmicutes* and *Patescibacteria* and decreased *Proteobacteria* in MAFLD rats. At the genus level, *Lactobacillus, Bacillus, Lachnospiraceae_NK4A136_group*, and *Dubosiella* increased, and *Escherichia‐Shigella* and *Blautia* reduced. Specifically, gut microbiota dysbiosis, characterized by an increased abundance of *Proteobacteria* and *Actinobacteria* and a decreased abundance of *Firmicutes*, reduces the expression of tight junction proteins. This directly impairs intestinal barrier function, allowing harmful microorganisms to pass through and stimulate the immune system, leading to immune cell inflammation and ultimately accelerating MAFLD (L. R. Zhu et al. 2023). *Lactobacillus* and *Desulfovibrio* are beneficial microorganisms that mitigate MAFLD by producing SCFAs, and higher levels of *Lactobacillaceae* in the gut are associated with lower levels of AST and ALT in the liver (L. R. Zhu et al. 2023). Additionally, the Lachnospiraceae_NK4A136_group produces butyrate, which helps maintain the integrity of the intestinal barrier in mice (Wan et al. 2022). Our study also demonstrated that *Lactobacillus, Lachnospiraceae_NK4A136_group*, and *Desulfovibrio* showed a negative association with MAFLD which is consistent with previous studies, while HQGG can improve the abundance of these gut microbiota, regulate gut microbiota imbalance, and exert anti‐MAFLD effect.

## 5 Conclusion

This research conducted network pharmacology and in vivo experiments to uncover the mechanisms of HQGG as a treatment for MAFLD. We found a variety of active components (such as quercetin, kaempferol, formononetin, puerarin, etc.) in HQGG, which act on multiple different targets through multiple biological processes and pathways to treat MAFLD. Validation experiments showed that HQGG may treat MAFLD by activating PPAR‐γ, which inhibits NF‐κB activity and significantly reduces the inflammatory response, thereby alleviating liver inflammation. At the same time, the gut microbiota analysis results indicated that HQGG could modulate the species structure and abundance of MAFLD rats. To our knowledge, this is the first instance revealing the mechanism of HQGG in treating MAFLD from the perspective of network pharmacology and gut microbiota, providing preliminary experimental evidence for the potential development and application of HQGG as functional food for the prevention of MAFLD.

## Author Contributions

**Jing Zhou:** funding acquisition (equal), resources (supporting), supervision (supporting), writing – review and editing (supporting). **Xianqiang Shao:** conceptualization (supporting), investigation (supporting), project administration (supporting), resources (supporting), supervision (supporting). **Shanshan Lei** and **Linzi Li:** conceptualization (equal), data curation (equal), formal analysis (equal), project administration (equal), resources (supporting), supervision (equal), visualization (equal), writing – review and editing (equal). **Yan Yang:** data curation (equal), formal analysis (equal), methodology (equal), software (equal), validation (equal), visualization (equal). **Cong Zhou:** data curation (supporting), formal analysis (supporting), methodology (equal), visualization (supporting). **Yuxian Zhang:** conceptualization (equal), data curation (equal), formal analysis (equal), project administration (equal). **Xu Ai:** data curation (supporting), formal analysis (supporting), funding acquisition (supporting), investigation (supporting), resources (supporting), supervision (supporting). **Daoping Zhang:** conceptualization (supporting), project administration (supporting), resources (supporting), supervision (supporting). **Chunkai Rao:** conceptualization (supporting), methodology (supporting), project administration (supporting), supervision (supporting). **Chengxiong Yang:** conceptualization (supporting), project administration (supporting), resources (supporting), supervision (supporting).

## Conflicts of Interest

The authors declare no conflicts of interest.

## Data Availability

The datasets used and/or analyzed during the current study are available from Dr. Lin‐zi Li on reasonable request.
